# Contact load is associated with both contact and non-contact injuries in rugby union

**DOI:** 10.3389/fphys.2025.1672824

**Published:** 2025-09-30

**Authors:** Yusuke Iwasaki, Yuki Someya, Masashi Nagao, Masashi Aoyagi, Yuki Shiota, Yuji Takazawa

**Affiliations:** ^1^ Graduate School of Health and Sports Science, Juntendo University, Chiba, Japan; ^2^ Department of Sports Medicine, Juntendo University, Tokyo, Japan; ^3^ Innovative Medical Technology Research and Development Center, Juntendo University, Tokyo, Japan; ^4^ Institute of Health and Sports Science and Medicine, Juntendo University, Chiba, Japan

**Keywords:** contact sports, injury prevention, non-contact injury, training load, loadmanagement, contact load, wearable technology

## Abstract

**Objective:**

Managing matches and training loads is crucial for injury prevention. Contact load is a defining feature of rugby union, and World Rugby has proposed its management as a key strategy for the prevention of injuries. In fact, increased contact load has been associated with a higher incidence of injuries. However, the specific relationship between contact load and the occurrence of both contact and non-contact injuries remains unclear. In this study, we aimed to clarify the association between contact load and the occurrence of contact and non-contact injuries in elite rugby union players.

**Methods:**

Sixty-six elite male rugby union players (age: 26.5 ± 3.5 years) in Japan were monitored over three seasons. Contact load, an indicator of training load, was evaluated based on collision count and collision load, measured using a global positioning system device. For each player, cumulative contact loads were calculated using time windows of 1, 2, 3, 4, 5, 6, 7, 14, 21, and 28 days. The association between contact load and injury incidence (contact and non-contact) was analyzed using generalized estimating equations.

**Results:**

A total of 193 injuries were recorded. Of these, 136 were contact injuries and 57 were non-contact injuries. The contact load was significantly associated with both types of injury. For contact injuries, the highest odds ratio for the collision count was observed on day 1 and gradually decreased toward day 7 (day 1: odds ratio, 2.10 [95% confidence interval: 1.67–2.64]; day 7: 1.31 [1.15–1.48]). The odds ratio for collision load also declined from days 1–7 (day 1: 3.27 [2.18–4.90]; day 7: 1.44 [1.17–1.78]). By contrast, non-contact injuries showed a different pattern. For collision count, the highest odds ratio was observed on day 2 and then gradually decreased toward day 4 (day 2: 1.38 [1.04–1.83]; day 4: 1.35 [1.06–1.72]). The odds ratio for collision load was also the highest on day 2 and decreased toward day 4 (day 2: 1.75 [1.16–2.65]; day 4: 1.56 [1.07–2.27]).

**Conclusion:**

Contact load was associated with both contact and non-contact injuries in elite rugby union players.

## 1 Introduction

Rugby union is a full-contact sport played worldwide (Duthie et al., 2005). It has one of the highest injury incidence rates among all professional team sports, with 91 and 2.8 injuries per 1,000 player-hours during matches and training, respectively (Williams et al., 2022). According to the mechanism of injury, most injuries can be classified as either contact or non-contact, with the exception of certain injuries. Contact injuries are most often caused by collisions, such as tackles during matches, and account for more than 60% of all reported injuries (Williams et al., 2022). Conversely, non-contact injuries without direct physical collisions occur more frequently during training sessions (Fuller et al., 2020). While contact injuries often involve accidental or unpredictable events and may be difficult to prevent entirely, non-contact injuries are considered preventable, as they are associated with modifiable risk factors such as aerobic capacity, strength, neuromuscular control, and tissue resilience (Meeuwisse et al., 2007; Gabbett, 2016). Therefore, to reduce the overall incidence of injuries in rugby union, it is essential not only to address contact injuries but also to focus on non-contact injuries by addressing these factors (Meeuwisse et al., 2007; Gabbett, 2016).

One widely used method for monitoring and managing injury risk is the use of Global Positioning System (GPS) devices, which track players’ physical loads during training and matches (Soligard et al., 2016; Windt and Gabbett, 2017; Andrade et al., 2020; Griffin et al., 2020; Maupin et al., 2020). In other field sports, such as soccer, a high running load has been linked to an increased incidence of non-contact injuries, particularly hamstring strains (Malone et al., 2017; Malone et al., 2017; Roe et al., 2018; Gómez-Piqueras and Alcaraz, 2024). Intense running loads are considered a primary factor in non-contact injuries, and sudden increases in high-speed running distance may increase the risk of non-contact injuries (Jaspers et al., 2018; Malone et al., 2018). Although rugby union teams have also used GPS technology to monitor non-contact variables such as overall distance (Cousins et al., 2019; Taylor et al., 2021; Ren et al., 2024), rugby union involves a shorter total running distance and less high-speed running compared to other field sports. In addition, rugby union features frequent and high-intensity contact plays instead of extensive high-speed running (Paul et al., 2022). World Rugby, the international governing body of rugby union, has proposed managing and limiting contact practice by monitoring “contact load” from the perspective of injury prevention (Starling et al., 2023). This guideline indicates that “contact load” comprises elements such as intensity (the magnitude of contact events), volume (the total amount of contact), density (the frequency of contacts), and unpredictability (the degree to which a player can anticipate their direct opponent’s actions during contact activities). Although contact is a defining feature of rugby union, the relationship between “contact load” and injury risk has not yet been fully clarified. Additionally, a 2023 systematic review of training loads in rugby football players reported a strong relationship between training loads and each athlete’s capacity for and tolerance of those loads. However, contact load has not yet been systematically quantified as a component of training load, the relationship between contact load, physical performance, and physiological adaptations in rugby players (Paiva et al., 2023).

Recent technological advancements have enabled some GPS devices to measure not only running metrics but also contact-related variables, such as contact intensity and volume (MacLeod et al., 2018; Tierney et al., 2020). Our previous study showed that higher “contact load”, as calculated using these GPS devices, was associated with an increase in the overall incidence of injuries (Iwasaki et al., 2024). However, the specific relationship between GPS-measured “contact load” and the mechanism of injury, whether contact or non-contact, remains unclear. Therefore, we aimed to investigate the association between “contact load” and the occurrence of both contact and non-contact injuries in elite rugby union players.

## 2 Materials and methods

### 2.1 Study design

In this retrospective observational study, we used load data from GPS devices and injury records of 66 elite male rugby union players. The participants were rugby union players who belonging to and playing in Japan Rugby League One, the highest level of rugby union league in Japan recognized by World Rugby. All participants were informed of the purpose, methods, procedures, and risks of the study and were provided with an opportunity to opt out. This study was conducted in accordance with the Declaration of Helsinki and was approved by the Ethics Committee for Human Experiments of Juntendo University (No. 2023-58). The observation period covered three rugby seasons, from 30 August 2021 to 25 May 2024.

### 2.2 Load data in matches and training

Load data during matches and field-based training sessions were obtained using a GPS device (STATSports Apex, Northern Ireland) (Beato et al., 2018; Thornton et al., 2019). This device collected data from a GPS, accelerometer, magnetometer, and gyroscope at frequencies of 10, 952, 10, and 952 Hz, respectively. The participants wore a specially designed vest which placed the GPS device on the upper back, that is, over the thoracic spine, between the left and right scapulae and the same device during the study to eliminate inter-unit variability and errors. Then, several load indicators were calculated using STATSports Sonra (STATSports): collision count, collision load, distance, and high-speed running. Collision was detected when the GPS device registered an impact greater than 8 g on the wearer’s body, accompanied by a shift in axial load direction. Collision count represents the frequency of collision events, and collision load is a composite metric representing the cumulative intensity of these events. Collision load metric is calculated using a proprietary weighted algorithm that combines the maximum velocity into the collision, peak impact force, and collision duration (MacLeod et al., 2018). In this study, collision count and collision load were quantified as “contact load”. Distance and high-speed running data were collected using GPS at a 10 Hz rate; high-speed running was defined as the distance covered at speeds >5.5 m/s (Beato et al., 2018).

### 2.3 Data processing and missing data

Collision count, collision load, distance, and high-speed running were used as load indicators for each participant during the matches and training. For each player, cumulative loads were calculated using time windows of 1, 2, 3, 4, 5, 6, 7, 14, 21, and 28 days for collision count, collision load, total distance, and high-speed running distance. Missing GPS data were imputed using the mean value from players in the same positional group (forward or back) for each session. This accounts for the varying training loads among positions in rugby union and was accomplished by adapting the Daily Team Mean (DTMean) method (Griffin et al., 2021).

### 2.4 Definition of injury

Injury was defined as physical discomfort that occurred during training or a match that prevented full participation in a training session or match. Injuries were diagnosed and classified by the team medical staff according to the 2007 consensus statement of the International Rugby Board (Fuller et al., 2007). Furthermore, the severity (number of days unavailable for training and/or matches), mechanism of injury (contact or non-contact), session in which the injury occurred (training or match), and type of injury were categorized as previously reported (Fuller et al., 2007). In accordance with the 2007 consensus statement, contact injuries were defined as those arising from contact with another player or object at the moment of injury. Any instances of indirect contact (e.g., a tackle to the upper body causing a knee ligament injury from the resulting twist) were also classified as contact injuries, as the statement does not differentiate a separate “indirect contact” category. Non-contact injuries were defined as those occurring in the absence of direct physical contact at the moment of injury. To reach a consensus on classification, all injury diagnoses and mechanisms were reviewed by at least two members of the medical team.

### 2.5 Statistical analysis

Odds ratios with 95% confidence intervals (CI) were calculated using multiple logistic regression analysis to determine the association between each load metric (collision count, collision load, distance, and high-speed running) across various time windows (1, 2, 3, 4, 5, 6, 7, 14, 21, and 28 days) and injury occurrence. As this study included repeated matches and training load data during the observation period, generalized estimating equations (GEE) were used to model the population-averaged effects of all data. First, athletes were treated as the subject variable, with the date of measurement as the within-subject variable, to account for the correlation between repeated injury incidence observations within subjects, using an autoregressive correlation matrix. The calculated model included injury occurrence (injury/no injury) as the dependent variable; each load metric within each time window as the independent variable; and position (forward/back), season (2021–2022/2022–2023/2023–2024), and age as confounders. All statistical analyses were performed using SPSS Statistics version 25 (IBM, Armonk, NY, USA), with statistical significance set at *P* < 0.05.

## 3 Results

All data from the 66 male elite rugby union players (36 forwards and 30 backs) included in the study were used (mean [SD], age: 26.5 [3.5] years, height: 181.0 [7.9] cm, weight: 98.7 [12.4] kg). The demographic characteristics of the participants are presented in Table 1. The number and types of injuries are shown in Tables 2, 3, respectively. During the cumulative observation period of 36,547 player-days, 193 injuries occurred (6.26 injuries/1000 player-hours), including 136 (70.5%) contact injuries and 57 (29.5%) non-contact injuries. In total, the cumulative number of days lost was 4,465 (12.2%). Of the 136 contact injuries, 75.0% (102 cases) occurred during matches, and 64.9% (37 out of 57) of the non-contact injuries occurred during training. Muscle and tendon injuries were the most common (74 cases) and accounted for 84.2% of non-contact injuries. By contrast, 69.1% of contact injuries occurred in the joint (non-bone)/ligament and brain/central peripheral nervous system.

**TABLE 1:** Demographic details of the study participants.

	Total (n = 66)	Forwards (n = 36)	Backs (n = 30)
Age, years	26.5 (3.5)	26.5 (3.4)	26.5 (3.6)
21–25	30 (45.5%)	16 (53.3%)	14 (46.7%)
26–30	27 (40.9%)	16 (59.3%)	11 (40.7%)
31–35	9 (13.6%)	4 (44.4%)	5 (55.6%)
Height, cm	181.0 (7.9)	183.8 (8.7)	177.6 (5.0)
Body weight, kg	98.7 (12.4)	107.7 (7.5)	88.0 (7.6)

Data are expressed as number (%) or mean (standard deviation).

**TABLE 2:** Total number and mechanism of injuries according to session type.

	Total	Injury/1000 player-hours (95% CI)	In matches	In training
Total number of injuries	193	6.26 (5.4–7.1)	122 (63.2%)	71 (36.8%)
Contact injuries	136	4.41 (3.7–5.1)	102 (75.0%)	34 (25.0%)
Non-contact injuries	57	1.85 (1.4–2.3)	20 (35.1%)	37 (64.9%)

Data are expressed as number (%) or median (95% CI).

**TABLE 3:** Type of injuries according to the mechanism of injuries.

Main group	Category	Total (n = 193, %)	Contact (n = 136, %)	Non-contact (n = 57, %)
Bone	All injuries	16 (8.3%)	15 (11.0%)	1 (1.8%)
	Fracture	15 (7.8%)	15 (11.0%)	0 (0.0%)
	Other bone injuries	1 (0.5%)	0 (0.0%)	1 (1.8%)
Joint (non-bone)/ligament	All injuries	68 (35.2%)	60 (44.1%)	8 (14.0%)
	Dislocation/subluxation	3 (1.6%)	3 (2.2%)	0 (0.0%)
	Sprain/ligament injury	49 (25.4%)	46 (33.8%)	3 (5.3%)
	Lesion meniscus/cartilage/disc	16 (8.3%)	11 (8.1%)	5 (8.8%)
Muscle/tendon	All injuries	74 (38.3%)	26 (19.1%)	48 (84.2%)
	Muscle tear/strain/cramps	56 (29.0%)	10 (7.4%)	46 (80.7%)
	Tendon injury/rupture/tendinopathy/bursitis	5 (2.6%)	3 (2.2%)	2 (3.5%)
	Hematoma/contusion/bruise	13 (6.7%)	13 (9.6%)	0 (0.0%)
Skin	All injuries	1 (0.5%)	1 (0.7%)	0 (0.0%)
	Laceration	1 (0.5%)	1 (0.7%)	0 (0.0%)
Brain/CPNS	All injuries	34 (17.6%)	34 (25.0%)	0 (0.0%)
	Concussion	29 (15.0%)	29 (21.3%)	0 (0.0%)
	Nerve injury	5 (2.6%)	5 (3.7%)	0 (0.0%)

Data are expressed as numbers (%). Percentages for “Total,” “Contact,” and “Non-Contact” columns are calculated out of 193, 136, and 57 injuries, respectively.CPNS, Central Peripheral Nervous System (spinal cord/peripheral nervous system).

### 3.1 Association between load by time window and injury

**FIGURE 1:**
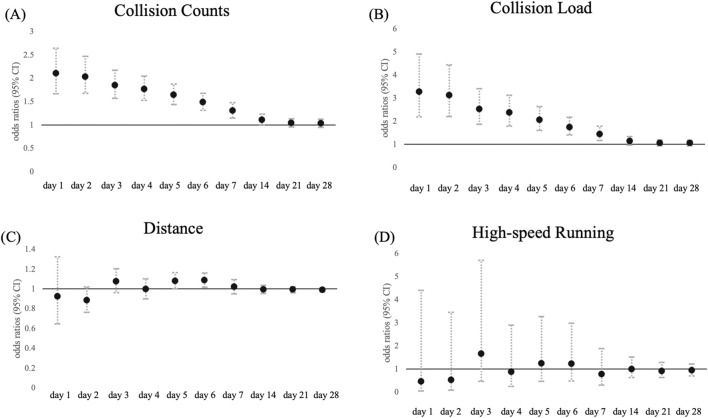
Association between match and training load at each time window and total injuries. Data are presented as odds ratio (points) and 95% confidence intervals (solid line) for the incidence of total injuries in each time window for **(A)** collision count, **(B)** collision load, **(C)** total distance, and **(D)** high-speed running.

The odds ratios of injuries associated with each load by time window are shown in Figure 1; Supplementary Table S1. Injuries were significantly associated with both collision count (Figure 1A) and collision load (Figure 1B) from days 1–7 (p < 0.01, respectively). For the collision count, the highest odds ratios were observed on day 1, and the odds ratio gradually decreased toward day 7 (day 1: odds ratio, 2.00 [95% CI: 1.57–2.54], day 7: 1.27 [1.15–1.48]). For collision load, the odds ratio also decreased from days 1–7 (day 1: 2.99 [1.98–4.54], day 7: 1.38 [1.17–1.78]). Regarding distance (Figure 1C), a weak but significant association was observed on day 6. However, these associations were not observed for high-speed running (Figure 1D). After day 14, no significant associations were observed between any of the load variables and injuries.

### 3.2 Association between load and contact or non-contact injuries

**FIGURE 2:**
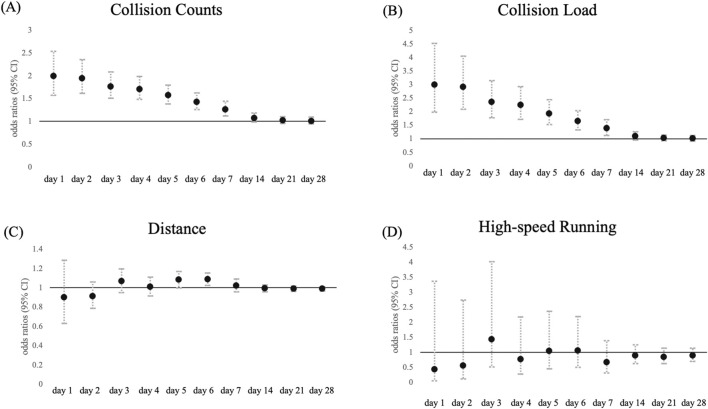
Association between match and training load at each time window and contact injuries. Data are presented as odds ratio (points) and 95% confidence intervals (solid line) for the incidence of contact injuries in each time window for **(A)** collision count, **(B)** collision load, **(C)** total distance, and **(D)** high-speed running.

**FIGURE 3:**
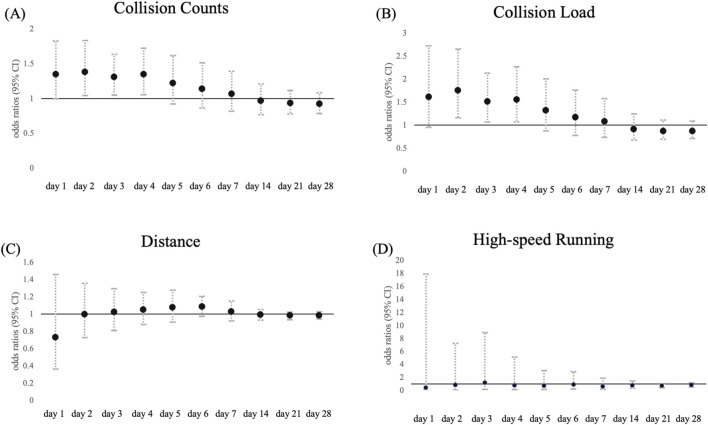
Association between match and training load at each time window and non-contact injuries. Data are presented as odds ratio (points) and 95% confidence intervals (solid line) for the incidence of non-contact injuries in each time window for **(A)** collision count, **(B)** collision load, **(C)** total distance, and **(D)** high-speed running.

The odds ratios of contact injuries associated with each load by time window are shown in Figure 2; Supplementary Table S2, whereas those of non-contact injuries are presented in Figure 3; Supplementary Table S3. The pattern of contact injuries largely matched that of overall injuries. For the collision count (Figure 2A), the highest odds ratio was observed on day 1, then gradually decreased toward day 7 (day 1: odds ratio, 2.10 [1.67–2.64], day 7: 1.31 [1.15–1.48]). For collision load (Figure 2B), the odds ratio also declined from days 1–7 (day 1: 3.27 [2.18–4.90], day 7: 1.44 [1.17–1.78]). No significant associations were observed after day 14. Although distance (Figure 2C) showed a weak but significant link on day 6 (1.08 [1.01–1.16]), high-speed running (Figure 2D) was not associated with contact injuries at any point. By contrast, non-contact injuries showed a different pattern. For collision count (Figure 3A), the highest odds ratio was observed on day 2, gradually decreasing toward day 4 (day 2: 1.38 [1.04–1.83], day 4: 1.35 [1.06–1.72]). For collision load (Figure 3B), the odds ratio was also the highest on day 2 and decreased toward day 4 (day 2: 1.75 [1.16–2.65], day 4: 1.56 [1.07–2.27]). No significant associations were found after day 5. Neither total distance (Figure 3C) nor high-speed running (Figure 3D) was associated with noncontact injuries in any time window.

## 4 Discussion

In this study, we investigated the association between “contact load” calculated using contact intensity and volume, and the occurrence of injuries in elite rugby union players. A total of 136 contact injuries and 57 non-contact injuries occurred during the study, both of which were significantly associated with “contact load,” regardless of injury type. In recent years, World Rugby has emphasized managing training load as a key strategy for player welfare and injury prevention. Specifically, they have developed and recommended the use of guidelines for monitoring and managing the “contact load” (Starling et al., 2023). Our previous study demonstrated that an increased acute:chronic workload ratio (ACWR) of “contact load” is associated with injury risk (Iwasaki et al., 2024). In this study, we also showed that “contact load” was associated with the occurrence of both contact and non-contact injuries in elite rugby union players. Our findings provide additional evidence supporting the importance of monitoring “contact load” for predicting and preventing all rugby union injuries.

One of the most important findings of this study is that “contact load” was associated not only with contact injuries but also with non-contact injuries. Among the 57 non-contact injuries identified, 84.2% involved muscle or tendon injuries. It has been reported that accumulated “contact load” may cause muscle damage, neuromuscular fatigue, and decreased performance (Takarada, 2003; Johnston et al., 2014; Naughton et al., 2018). For example, Takarada found that players performing more tackles had higher myoglobin and creatine kinase (CK) levels 24 h post-match (Takarada, 2003), whereas Johnston et al. showed that greater volumes of full-contact tackling elevated CK and reduced upper body neuromuscular function (Johnston et al., 2014). Similarly, Naughton et al. observed that sprint times and jump heights remained impaired for 48–72 h following contact play (Naughton et al., 2018). Such temporary increases in muscle damage and decreases in muscle strength, speed, and jump performance over several days may negatively affect modifiable risk factors (e.g., aerobic capacity, strength, neuromuscular control, and tissue resilience) and potentially increase the risk of non-contact injuries (Windt and Gabbett, 2017). However, running loads such as running distance and high-speed running were not clearly associated with non-contact injuries in this study. This finding is inconsistent with those of previous studies on soccer, which suggested that high running loads are linked to an increased incidence of non-contact injuries, particularly hamstring strains (Malone et al., 2017; Malone et al., 2017; Roe et al., 2018; Gómez-Piqueras and Alcaraz, 2024). Professional soccer players typically cover approximately 10,000–13,000 m per match, of which approximately 1,000–1,200 m are run at speeds exceeding 5.5 m/s (high-speed running) (Barnes et al., 2014). By contrast, professional rugby union players typically cover approximately 5,000–7,500 m in total, with backs running approximately 300–600 m of high-speed running and forwards running approximately 100–300 m (Quarrie et al., 2013; Reardon et al., 2015). Thus, professional rugby union players might not reach the threshold for increased injury risk. In addition, rugby union features frequent and high-intensity contact play, instead of extensive high-speed running (Paul et al., 2022). Therefore, these sport-specific demands suggest that in elite rugby union, “contact load” might be a more significant risk factor for non-contact injuries than running load.

We also showed that contact injuries were strongly associated with “contact load” from day 1 (the day of injury) through day 7, with the highest odds ratio on day 1. Although this odds ratio gradually decreased on each subsequent day, it remained significant until day 7. On the other hand, non-contact injuries showed a significant relationship with “contact load” from days 2–4, the odds ratio on day 1 was not highest. Duration of acute load “time window” is defined as the load within 7 days according to the IOC (Impellizzeri et al., 2020), and a 7-day time window is generally recognized and widely used. However, there is a lack of clear scientific evidence supporting the optimality of this 7-day window, and the appropriate time window may vary depending on the sport and schedule (Impellizzeri et al., 2020). The findings of this study align with those of previous studies in that shorter time windows can more effectively capture injury risk (Carey et al., 2017; West et al., 2021). However, as not all injuries may be caused solely by loads within these time windows, and some may result from more chronic load accumulation. Therefore, future research using analytical approaches that consider sport-specific, schedule-specific and athlete factors need to clarify more optimal time windows for injury prevention.

This study has several limitations. First, collision events were detected using a GPS device based on an algorithm that incorporates changes in axis orientation and impacts exceeding 8 g; however, the exact details of this proprietary algorithm are not publicly available. Therefore, specific collision characteristics (such as the direction of impact) and compare with contact lad using different technologies or GPS device cannot be evaluated. Further discussion is needed on the detailed interpretation of “contact load”. Second, the load data were presented as absolute value and have not been normalized according to the duration of individual training or match. In order to provide injury risk assessments based on load relative to practice or match duration, the data may need to be normalized based on exposure time. Third, because only external loads that can be measured by GPS were considered, indoor sessions such as gym training were not included. Fourth, although we adjusted for basic confounding factors, such as playing position, season, and age, other potential risk factors, such as injury history, internal load (e.g., session rating of perceived exertion or subjective fatigue), individual recovery practices, technical skills, and body composition were not considered and may also influence injury risk. Finally, because this was an observational study and injury incident has various factor, a direct causal link between “contact load” management and a reduction in injury incidence cannot be established. Therefore, intervention-based research or observational studies that include multiple factors are needed to verify this causal relationship.

In conclusion, our study showed that “contact load” is associated with not only contact injuries but also non-contact injuries in elite rugby union players. The monitoring and management of “contact load” should be a key consideration in reducing injury risk and enhancing performance.

## Data Availability

The original contributions presented in the study are included in the article/Supplementary Material, further inquiries can be directed to the corresponding author.

## Abbreviations

GPS, Global Positioning System; CI, confidence interval; GEE, generalized estimating equation; ACWR, acute chronic workload ratio; CK, creatine kinase.
