# Supplementary material for: Contact load is associated with both contact and non-contact injuries in rugby union
Source: Front Physiol. 2025 Sep 30;16:1672824. doi: 10.3389/fphys.2025.1672824 (PMC12518334; doi:10.3389/fphys.2025.1672824)
Supplement: Supplementary file 1 [file Table1.docx]

Supplementary Material

# Supplementary Tables

**Supplementary Table 1.** Association between match and training loads at each time window and total injuries

|  | Time  Window | Intercept | Parameter | Std. error | Odds  ratio | 95% CI |
| --- | --- | --- | --- | --- | --- | --- |
|  |  |  | estimate |  |  |  |
| Collision counts | day 1 | -7.63 | 0.69 | 0.12 | 2.00 | 1.57-2.54 |
|  | day 2 | -7.67 | 0.67 | 0.10 | 1.95 | 1.61-2.36 |
|  | day 3 | -7.71 | 0.57 | 0.08 | 1.77 | 1.51-2.09 |
|  | day 4 | -7.82 | 0.54 | 0.08 | 1.72 | 1.48-1.99 |
|  | day 5 | -7.82 | 0.46 | 0.07 | 1.58 | 1.39-1.80 |
|  | day 6 | -7.76 | 0.36 | 0.06 | 1.43 | 1.26-1.62 |
|  | day 7 | -7.60 | 0.24 | 0.07 | 1.27 | 1.12-1.44 |
|  | day 14 | -7.42 | 0.08 | 0.05 | 1.08 | 0.99-1.19 |
|  | day 21 | -7.29 | 0.03 | 0.04 | 1.03 | 0.95-1.11 |
|  | day 28 | -7.38 | 0.01 | 0.04 | 1.01 | 0.94-1.09 |
| Collision load | day 1 | -7.61 | 1.10 | 0.21 | 2.99 | 1.98-4.52 |
|  | day 2 | -7.70 | 1.07 | 0.17 | 2.91 | 2.09-4.06 |
|  | day 3 | -7.68 | 0.86 | 0.15 | 2.36 | 1.77-3.14 |
|  | day 4 | -7.80 | 0.81 | 0.14 | 2.24 | 1.72-2.93 |
|  | day 5 | -7.77 | 0.66 | 0.12 | 1.93 | 1.53-2.44 |
|  | day 6 | -7.70 | 0.50 | 0.11 | 1.64 | 1.33-2.03 |
|  | day 7 | -7.52 | 0.33 | 0.11 | 1.38 | 1.13-1.70 |
|  | day 14 | -7.29 | 0.09 | 0.07 | 1.10 | 0.96-1.26 |
|  | day 21 | -7.14 | 0.02 | 0.05 | 1.02 | 0.92-1.14 |
|  | day 28 | -7.24 | 0.01 | 0.05 | 1.01 | 0.92-1.12 |
| Distance | day 1 | -6.58 | -0.11 | 0.18 | 0.90 | 0.63-1.28 |
|  | day 2 | -6.44 | -0.09 | 0.08 | 0.91 | 0.79-1.06 |
|  | day 3 | -7.67 | 0.06 | 0.06 | 1.06 | 0.95-1.19 |
|  | day 4 | -7.18 | 0.01 | 0.05 | 1.01 | 0.92-1.11 |
|  | day 5 | -8.06 | 0.08 | 0.04 | 1.08 | 0.99-1.17 |
|  | day 6 | -8.25 | 0.08 | 0.03 | 1.09 | 1.02-1.15 |
|  | day 7 | -7.44 | 0.02 | 0.03 | 1.02 | 0.96-1.09 |
|  | day 14 | -6.99 | -0.01 | 0.02 | 0.99 | 0.96-1.03 |
|  | day 21 | -6.69 | -0.01 | 0.01 | 0.99 | 0.96-1.01 |
|  | day 28 | -6.63 | -0.01 | 0.01 | 0.99 | 0.97-1.01 |
| High-speed running | day 1 | -7.11 | -0.88 | 1.07 | 0.41 | 0.05-3.36 |
|  | day 2 | -6.94 | -0.61 | 0.82 | 0.54 | 0.11-2.74 |
|  | day 3 | -7.54 | 0.36 | 0.53 | 1.43 | 0.51-4.01 |
|  | day 4 | -7.27 | -0.27 | 0.53 | 0.76 | 0.27-2.17 |
|  | day 5 | -7.42 | 0.03 | 0.43 | 1.03 | 0.45-2.36 |
|  | day 6 | -7.34 | 0.04 | 0.38 | 1.04 | 0.50-2.19 |
|  | day 7 | -7.02 | -0.42 | 0.38 | 0.66 | 0.31-1.38 |
|  | day 14 | -7.25 | -0.13 | 0.18 | 0.88 | 0.62-1.25 |
|  | day 21 | -7.12 | -0.18 | 0.16 | 0.84 | 0.62-1.14 |
|  | day 28 | -7.33 | -0.12 | 0.12 | 0.89 | 0.70-1.13 |

CI, confidence interval.

**Supplementary Table 2.** Association between match and training loads at each time window and contact injuries

|  | Time  Window | Intercept | Parameter | Std. error | Odds  ratio | 95% CI |
| --- | --- | --- | --- | --- | --- | --- |
|  |  |  | estimate |  |  |  |
| Collision counts | day 1 | -7.84 | 0.74 | 0.12 | 2.10 | 1.67-2.64 |
|  | day 2 | -7.95 | 0.71 | 0.10 | 2.03 | 1.68-2.46 |
|  | day 3 | -7.97 | 0.61 | 0.08 | 1.85 | 1.57-2.17 |
|  | day 4 | -8.06 | 0.57 | 0.07 | 1.77 | 1.53-2.05 |
|  | day 5 | -8.09 | 0.50 | 0.07 | 1.64 | 1.44-1.88 |
|  | day 6 | -8.04 | 0.40 | 0.06 | 1.49 | 1.31-1.68 |
|  | day 7 | -7.87 | 0.27 | 0.06 | 1.31 | 1.15-1.48 |
|  | day 14 | -7.60 | 0.10 | 0.05 | 1.11 | 1.00-1.23 |
|  | day 21 | -7.48 | 0.04 | 0.04 | 1.04 | 0.96-1.13 |
|  | day 28 | -7.49 | 0.03 | 0.04 | 1.03 | 0.95-1.12 |
| Collision load | day 1 | -7.80 | 1.19 | 0.21 | 3.27 | 2.18-4.90 |
|  | day 2 | -7.97 | 1.14 | 0.18 | 3.13 | 2.20-4.44 |
|  | day 3 | -7.92 | 0.93 | 0.15 | 2.52 | 1.88-3.40 |
|  | day 4 | -8.03 | 0.86 | 0.14 | 2.36 | 1.79-3.12 |
|  | day 5 | -8.02 | 0.72 | 0.13 | 2.06 | 1.61-2.63 |
|  | day 6 | -7.96 | 0.56 | 0.11 | 1.74 | 1.40-2.17 |
|  | day 7 | -7.78 | 0.37 | 0.11 | 1.44 | 1.17-1.78 |
|  | day 14 | -7.47 | 0.13 | 0.08 | 1.14 | 0.97-1.33 |
|  | day 21 | -7.32 | 0.05 | 0.06 | 1.05 | 0.93-1.19 |
|  | day 28 | -7.33 | 0.04 | 0.06 | 1.04 | 0.93-1.17 |
| Distance | day 1 | -7.11 | -0.08 | 0.18 | 0.92 | 0.65-1.32 |
|  | day 2 | -6.67 | -0.13 | 0.07 | 0.88 | 0.76-1.02 |
|  | day 3 | -8.07 | 0.07 | 0.06 | 1.07 | 0.96-1.20 |
|  | day 4 | -7.42 | 0.00 | 0.05 | 1.00 | 0.90-1.10 |
|  | day 5 | -8.39 | 0.08 | 0.04 | 1.08 | 1.00-1.16 |
|  | day 6 | -8.60 | 0.08 | 0.03 | 1.08 | 1.01-1.16 |
|  | day 7 | -7.74 | 0.02 | 0.04 | 1.02 | 0.95-1.09 |
|  | day 14 | -7.25 | -0.01 | 0.02 | 0.99 | 0.95-1.04 |
|  | day 21 | -7.04 | -0.01 | 0.02 | 0.99 | 0.96-1.02 |
|  | day 28 | -6.86 | -0.01 | 0.01 | 0.99 | 0.97-1.01 |
| High-speed running | day 1 | -7.46 | -0.82 | 1.17 | 0.44 | 0.05-4.39 |
|  | day 2 | -7.29 | -0.66 | 0.97 | 0.52 | 0.08-3.45 |
|  | day 3 | -7.84 | 0.50 | 0.64 | 1.64 | 0.47-5.71 |
|  | day 4 | -7.54 | -0.15 | 0.62 | 0.86 | 0.25-2.90 |
|  | day 5 | -7.77 | 0.21 | 0.50 | 1.23 | 0.46-3.26 |
|  | day 6 | -7.71 | 0.19 | 0.46 | 1.21 | 0.49-2.98 |
|  | day 7 | -7.28 | -0.28 | 0.46 | 0.76 | 0.30-1.88 |
|  | day 14 | -7.52 | -0.02 | 0.22 | 0.98 | 0.63-1.52 |
|  | day 21 | -7.43 | -0.11 | 0.18 | 0.90 | 0.63-1.29 |
|  | day 28 | -7.50 | -0.08 | 0.14 | 0.92 | 0.70-1.21 |

CI, confidence interval.

**Supplementary Table 3.** Association between match and training loads at each time window and noncontact injuries

|  | Time  Window | Intercept | Parameter | Std. error | Odds  ratio | 95% CI |
| --- | --- | --- | --- | --- | --- | --- |
|  |  |  | estimate |  |  |  |
| Collision counts | day 1 | -9.03 | 0.30 | 0.16 | 1.35 | 0.99-1.83 |
|  | day 2 | -8.87 | 0.32 | 0.14 | 1.38 | 1.04-1.83 |
|  | day 3 | -8.94 | 0.27 | 0.11 | 1.31 | 1.05-1.64 |
|  | day 4 | -9.06 | 0.30 | 0.12 | 1.35 | 1.06-1.72 |
|  | day 5 | -8.97 | 0.20 | 0.14 | 1.22 | 0.93-1.62 |
|  | day 6 | -8.89 | 0.13 | 0.14 | 1.14 | 0.86-1.51 |
|  | day 7 | -8.78 | 0.06 | 0.14 | 1.07 | 0.82-1.39 |
|  | day 14 | -8.77 | -0.04 | 0.12 | 0.96 | 0.77-1.21 |
|  | day 21 | -8.51 | -0.07 | 0.09 | 0.93 | 0.78-1.12 |
|  | day 28 | -8.78 | -0.08 | 0.08 | 0.92 | 0.79-1.08 |
| Collision load | day 1 | -9.04 | 0.48 | 0.27 | 1.61 | 0.95-2.72 |
|  | day 2 | -8.93 | 0.56 | 0.21 | 1.75 | 1.16-2.65 |
|  | day 3 | -8.95 | 0.41 | 0.18 | 1.51 | 1.07-2.13 |
|  | day 4 | -9.07 | 0.44 | 0.19 | 1.56 | 1.07-2.27 |
|  | day 5 | -8.95 | 0.28 | 0.21 | 1.32 | 0.87-2.00 |
|  | day 6 | -8.84 | 0.16 | 0.21 | 1.17 | 0.78-1.76 |
|  | day 7 | -8.73 | 0.08 | 0.19 | 1.08 | 0.74-1.58 |
|  | day 14 | -8.64 | -0.09 | 0.16 | 0.92 | 0.68-1.25 |
|  | day 21 | -8.38 | -0.13 | 0.12 | 0.88 | 0.69-1.11 |
|  | day 28 | -8.67 | -0.13 | 0.11 | 0.88 | 0.71-1.09 |
| Distance | day 1 | -6.92 | -0.32 | 0.35 | 0.73 | 0.37-1.46 |
|  | day 2 | -7.96 | -0.01 | 0.16 | 0.99 | 0.73-1.36 |
|  | day 3 | -8.65 | 0.02 | 0.12 | 1.02 | 0.81-1.29 |
|  | day 4 | -8.79 | 0.05 | 0.09 | 1.05 | 0.88-1.25 |
|  | day 5 | -9.25 | 0.07 | 0.09 | 1.07 | 0.91-1.27 |
|  | day 6 | -9.42 | 0.08 | 0.06 | 1.08 | 0.97-1.21 |
|  | day 7 | -8.81 | 0.03 | 0.06 | 1.03 | 0.92-1.15 |
|  | day 14 | -8.56 | -0.01 | 0.03 | 0.99 | 0.93-1.05 |
|  | day 21 | -7.91 | -0.02 | 0.02 | 0.98 | 0.93-1.03 |
|  | day 28 | -8.22 | -0.02 | 0.02 | 0.98 | 0.94-1.03 |
| High-speed running | day 1 | -8.33 | -0.89 | 1.93 | 0.41 | 0.01-17.86 |
|  | day 2 | -8.19 | -0.26 | 1.15 | 0.77 | 0.08-7.27 |
|  | day 3 | -8.92 | 0.16 | 1.04 | 1.17 | 0.15-8.89 |
|  | day 4 | -8.78 | -0.30 | 0.98 | 0.74 | 0.11-5.11 |
|  | day 5 | -8.65 | -0.35 | 0.75 | 0.71 | 0.16-3.06 |
|  | day 6 | -8.56 | -0.18 | 0.64 | 0.84 | 0.24-2.90 |
|  | day 7 | -8.55 | -0.59 | 0.63 | 0.55 | 0.16-1.90 |
|  | day 14 | -8.69 | -0.34 | 0.37 | 0.71 | 0.34-1.47 |
|  | day 21 | -8.42 | -0.37 | 0.23 | 0.69 | 0.44-1.09 |
|  | day 28 | -9.00 | -0.23 | 0.21 | 0.80 | 0.52-1.21 |

CI, confidence interval.
